# Cystic lymphangioma of the lesser sac presenting as acute appendicitis: A case report

**DOI:** 10.1186/1757-1626-1-147

**Published:** 2008-09-10

**Authors:** Benjamin HL Tan, Teegan Lim

**Affiliations:** 1 Tissue Injury and Repair Group, University of Edinburgh, 1st Floor Chancellor's Building, 49 Little France Crescent, Edinburgh, EH16 4SB, UK; 2 Department of Gastroenterology, Rochdale Infirmary, Whitehall Street, Rochdale, OL12 0NB, UK

## Abstract

Intra-abdominal lymphangiomas usually present by 2 years of age and are uncommon in adults. Cystic lymphagiomas arising from the lesser sac are even more uncommon. We report an unusual case of a lesser sac cystic lymphangioma presenting as acute appendicitis. A 21 year old female was admitted with pyrexia, right iliac fossa tenderness and an elevated C-reactive protein (CRP). At laparotomy, a large fluid filled cystic lesion was observed occupying the right side of the abdominal cavity. The lesion was excised in its entirety and histological diagnosis confirmed lymphangioma. The patient remains well with no evidence of recurrence 1 year post resection.

## Introduction

Lymphangiomas are rare, benign tumours of the lymphatic tissue, most commonly found in the neck. It usually presents in childhood, and most cases are seen within the first two years of life. Intra-abdominal lymphangiomas are not common [1]. Lymphangiomas of the lesser sac are even rarer and very few cases have been previously reported [2]. We report an unusual case of cystic lymphangioma of the lesser sac presenting as acute appendicitis in an adult female.

## Case report

A 21 year old female student was referred by her general practitioner with a three day history of central abdominal pain which moved to the right iliac fossa after two days. On examination, she was pyrexial with a temperature of 38.2°C and acutely tender in the right iliac fossa. Blood inflammatory markers revealed normal white cell counts but an elevated C-reactive protein (CRP) of 342 mg/l. She was admitted with the working diagnosis of acute appendicitis and a decision was made to carry out an emergency appendectomy.

Upon induction of anaesthesia, it was noted that there was a large mass within the abdomen. The mass extended from the epigastrium to the hypochondrium and was fluctuant. A midline laparotomy was subsequently performed.

**Figure 1:**
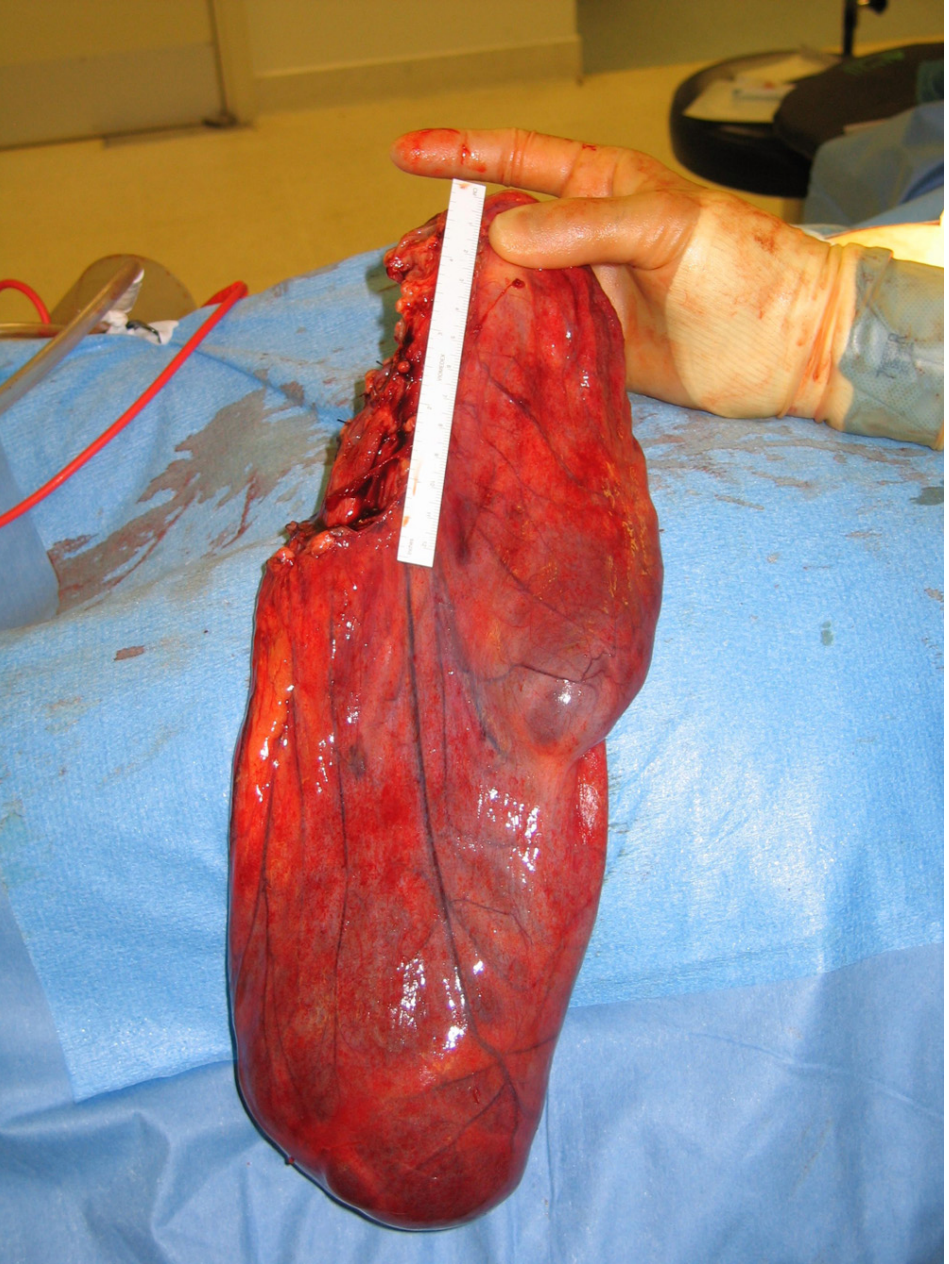
**Completely excised cyst.** White marker is 12 cm in length.

Upon entry into the abdominal cavity, it was noted that a large multi-loculated cyst was occupying most of the right side of the abdomen. On closer inspection, the cyst originted from the lesser sac and extended to the hypochondrium and right iliac fossa. The cyst had an extensive vascular supply and was filled with a foul smelling yellowish fluid. The cyst was completely excised and measured roughly 25 cm × 15 cm (Figure 1) and contained about 5 litres of fluid.

Histopathological examination of the cyst wall demonstrated loose fibrocellular connective tissue with prominent vasculature with areas of severe inflammation. On the basis of these findings, the cystic lesion was diagnosed as a benign cystic lymphangioma with inflammatory changes.

The patient remains well with no evidence of recurrence 1 year post resection.

## Discussion

Lymphangiomas are benign lesions of the lymphatic system whose exact aetiology remains uncertain. 90% of lymphangiomas occur in patients less than two years old, most commonly in the neck or axilla, in locations of primitive lymph sacs and are often referred to as 'cystic hygromas'. Intra-abdominal lymphangiomas are uncommon [1]. Lymphangiomas arising from the lesser sac are particularly unusual [2]. First presentation in adulthood is rare, but intra-peritoneal cystic lymphangiomas may present incidentally in later life, typically slowly enlarging and remaining asymptomatic for a long period although if large they can present with pressure effects on adjacent organs [3].

There have been no previous cases recorded in the literature of cystic lymphangiomas arising from the lesser sac presenting as acute appendicitis in an adult. The cause of this unusual presentation is likely infection of the contents of the cyst with subsequent inflammation.

Surgical excision is the optimal treatment for symptomatic lymphangiomas and often total excision of the lesion is readily possible with cystic lymphangiomas [4]. Excellent prognosis is achieved in complete resection with negative microscopic margins. However, if not excised completely, the intra-abdominal cystic lymphangioma has a 10% postoperative recurrence rate [5]. Rarely do lymphangiomas undergo malignant change or spontaneous regression [6].

In conclusion, cystic lymphangiomas arising from the lesser sac are rare tumours in adults with the potential to present acutely.

## Consent

Written informed consent was obtained from the patient for publication of this case report and accompanying images. A copy of the written consent is available for review by the Editor-in-Chief of this journal.

## Competing interests

The authors declare that they have no competing interests.

## Authors' contributions

BT was involved in the patient care, acquisition of data and writing of the manuscript. TL contributed to the writing of the manuscript. All authors read and approved the final manuscript.

## References

[B1] Enzinger FM, Weiss SW, Gay SM (1988). Tumors of lymph vessels. Soft tissue tumors.

[B2] Tezuka K, Ogawa Y, Satake K, Ohira M, Yamada S, Uno H, Wakasa K, Hirakawa K (2002). Lymphangioma of the lesser omentum associated with abdominal esophageal carcinoma: report of a case. Surg Today.

[B3] Taylor DH, Loughrey C (2004). Acute presentation of lymphangioma of the retroperitoneum. Ulster Med J.

[B4] Burkett JS, Pickleman J (1994). The rationale for surgical treatment of mesenteric and retroperitoneal cysts. Am Surg.

[B5] Steyaert H, Guitard J, Moscovici J, Juricic M, Vaysse P, Juskiewenski S (1996). Abdominal cystic lymphangioma in children: benign lesions that can have a proliferative course. J Pediatr Surg.

[B6] Henzel JH, Pories WJ, Burget DE, Smith JL (1966). Intra-abdominal lymphangiomata. Arch Surg.

